# TH2/TH1 Shift Under Ibrutinib Treatment in Chronic Lymphocytic Leukemia

**DOI:** 10.3389/fonc.2021.637186

**Published:** 2021-04-15

**Authors:** Maria Cristina Puzzolo, Ilaria Del Giudice, Nadia Peragine, Paola Mariglia, Maria Stefania De Propris, Luca Vincenzo Cappelli, Livio Trentin, Gianluigi Reda, Antonio Cuneo, Stefano Molica, Alfonso Piciocchi, Valentina Arena, Francesca Romana Mauro, Anna Guarini, Robin Foà

**Affiliations:** ^1^ Hematology, Department of Translational and Precision Medicine, Sapienza University, Rome, Italy; ^2^ Hematology Unit, University of Padua, Padua, Italy; ^3^ Hematology Unit, Fondazione IRCCS Cà Granda Ospedale Maggiore Policlinico, Milan, Italy; ^4^ Hematology Section, Department of Medical Science, Azienda Ospedaliero-Universitaria Arcispedale S. Anna, University of Ferrara, Ferrara, Italy; ^5^ Hematology, Azienda Ospedaliera “Pugliese Ciaccio”, Presidio Ospedaliero A. Pugliese - Unità Operativa di Ematologia, Catanzaro, Italy; ^6^ GIMEMA Data Center, GIMEMA Foundation, Rome, Italy; ^7^ Department of Molecular Medicine, Sapienza University, Rome, Italy

**Keywords:** chronic lymphocytic leukemia, ibrutinib, Th1, Th2, T lymphocytes

## Abstract

Ibrutinib may revert the T-helper (Th)2 polarization observed in chronic lymphocytic leukemia (CLL) by targeting the IL-2-inducible kinase, that shows a significant homology with the Bruton tyrosine kinase. In the front-line GIMEMA LLC1114 trial (ibrutinib+rituximab for 6 months, followed by ibrutinib maintenance), we investigated the modulation of T-cell cytokine production in 208 peripheral blood paired samples from 71 CLL patients: 71 samples prior to treatment (Day 0, D0) and at day +14 (D14; n=50), at month +8 (M8; 30), +12 (M12; 25), +18 (M18; 22) and +24 (M24; 10) of treatment. We documented a progressive decrease of CD3+CD4+IL-4+ T cells (Th2), that was significant at M8 and at M12 (p=0.019, p=0.002), a relative increase in the CD3+CD4+IFNγ+ T cells (Th1) and a decrease of CD3+CD4+IL-17+ (Th17) cells that was maintained up to M18 (M8 *vs* D0 p=0.003, M12 *vs* D0 p=0.003, M18 *vs* D0 p=0.004) of ibrutinib treatment. The Th2/Th1 ratio significantly decreased already after 14 days of treatment and was maintained thereafter (D14 *vs* D0 p=0.037, M8 *vs* D0 p=0.001, M12 *vs* D0 p=0.005, M18 *vs* D0 p=0.002). The Th2/Th1 modulation over time was significant only among patients with unmutated IGHV. The Th2/Th1 ratio below a cut-off of 0.088 at M8 was associated with the achievement of a complete response (CR) (p=0.016). Ibrutinib may shape the CLL T-cell profile, limiting Th2 activation and inducing a shift in the Th2/Th1 ratio. The association between the Th2/Th1 ratio decrease and the CR achievement suggests the *in vivo* generation of a potential host anti-tumor immune activation induced by ibrutinib.

## Introduction

In chronic lymphocytic leukemia (CLL), malignant B cells and the surrounding tissue microenvironment closely interact. The complex cross talk between normal and CLL cells plays a critical role in the survival, growth and drug resistance of leukemic cells (1–3). In addition, the leukemic clone develops strategies of evasion or suppression of the immune system, in particular toward the anti-tumor effects of T lymphocytes (4–7).

The T-cell compartment in CLL patients is dysfunctional, showing in particular: i) an increased expression of inhibitory receptors and a defective immune synapse formation upon contact with CLL cells (8); ii) skewing of T-cell subsets from naïve to memory T cells and an increase in the number of circulating T cells both in the CD4^+^ and the CD8^+^ compartments (9); iii) a higher expression of exhaustion markers (10); iv) within the CD4+ cells, a T-helper (Th)2/Th1 ratio imbalance with an aberrant recruitment of a Th2-dominant response (11, 12).

Indeed, CLL cells produce IL-6 and stimulate IL-4 production by T cells, skewing the immune system toward a Th2-phenotype, that releases IL-4, IL-5, IL-10 and IL-13 (13–16). Moreover, IL-10, a cytokine produced by Th2 cells, is a powerful inhibitor of the Th1 cytokine synthesis - including interferon γ (IFNγ), tumor necrosis factor α (TNFα), IL-2 and lymphotoxin (LTα) (14) - and stimulates B-cell proliferation and differentiation, thus promoting the skewing toward a Th2 response.

Finally, CLL cells also contribute to the imbalance between Th17 and T regulatory cells (T regs) by producing IL-10 or TGF-β, which promote the development of Tregs and suppress Th1, Th17 and cytotoxic T-cell responses. An increase in the number of Th17 cells (able to produce IL-17A and IL-17F) (17, 18) has been associated with an improved survival of CLL patients, while a decreased frequency of Th17 cells has been generally found to be associated with a Tregs expansion and disease progression (19).

The constitutive activation of the B-cell receptor (BCR) is central to CLL pathogenesis and prognosis. Among the new generation of biologic drugs, ibrutinib, an irreversible inhibitor of the Bruton tyrosine kinase (BTK), involved in the downstream pathway of the BCR signaling, has demonstrated outstanding clinical activity and tolerability in CLL (20). The IL-2-inducible kinase (ITK) is a T-cell dominant member of the TEC kinase family that drives proximal T-cell receptor (TCR) signaling, resulting in cellular activation, cytokine release and rapid proliferation (21–23). ITK plays a key role in the activation of Th1, Th2 and Th17 cells. In Th1 cells, ITK signaling is supportive but dispensable due to the redundant resting lymphocyte kinase (RLK) signaling, whereas in Th2 cells ITK signaling is essential for activation (23). The significant homology between BTK and ITK supports the role of ibrutinib as an immunomodulatory inhibitor of both BTK and ITK (24). Indeed, and at variance from chemoimmunotherapy, ibrutinib mitigates the immune dysregulation induced by CLL, by modifying the absolute number of T-cells (25–28), the T-cell receptor repertoire (26, 29), the T-cell maturation (27, 28), the Th1 and Th2 polarization (24–27), the Th17 and Treg cell balance (25, 27, 28), the T cell inhibitory receptors and function (25, 27–29). These effects, all modulated toward an anti-tumor T-cell response, have been nicely reviewed (30–32). Regarding the Th1 and Th2 polarization, it has been shown that ibrutinib may contribute to revert the Th2-dominant response observed in CLL, thus influencing T-cell mediated cancer immune surveillance, *in vitro* and in a mouse model (24) and in patients treated with ibrutinib (25–27). However, the variability of patients’ selection and of the experimental assays makes the results not always consistent. Differences between the reported *in vitro* and *in vivo* effects of ibrutinib therapy on Th polarization highlights the importance of correlative analysis in clinical trials.

In order to gain insights into the role of ibrutinib on the Th2/Th1 balance *in vivo*, we investigated the modulation of T-cell cytokine production in CLL patients enrolled in the ibrutinib + rituximab front-line GIMEMA LLC1114 trial.

## Materials and Methods

### Patients’ Samples

Cytokine production was evaluated in patients enrolled in the front-line GIMEMA LLC1114 trial (NCT02232386) for treatment-naive (TN) and unfit CLL patients requiring treatment according to the 2008 revised iwCLL criteria (33). The protocol was based on the administration of ibrutinib (420 mg/die) + rituximab (375 mg/m^2^), weekly in the first month and monthly from the 2^nd^ to the 6^th^ month) for 6 months, followed by ibrutinib maintenance, given up to 6 years or until disease progression, toxicity or undetectable minimal residual disease (MRD) for 6 months.

To follow intra-individual variations, Th-cell cytokine production was evaluated longitudinally in 71 patients, 25 females and 46 males; median age was 72.6 years (range 37.2-86.6). A total of 208 peripheral blood (PB) paired samples was included: 71 were collected prior to ibrutinib treatment (Day 0, D0), 50 after 14 days (D14), 30 after 8 months (M8), 25 after 12 months (M12), 22 after 18 months (M18) and 10 after 24 months (M24).

All patients gave a written informed consent for the research purposes according to the Declaration of Helsinki. The diagnosis of CLL was based on the current guidelines (33).

### Intracellular Cytokine Production Assay

**Figure 1:**
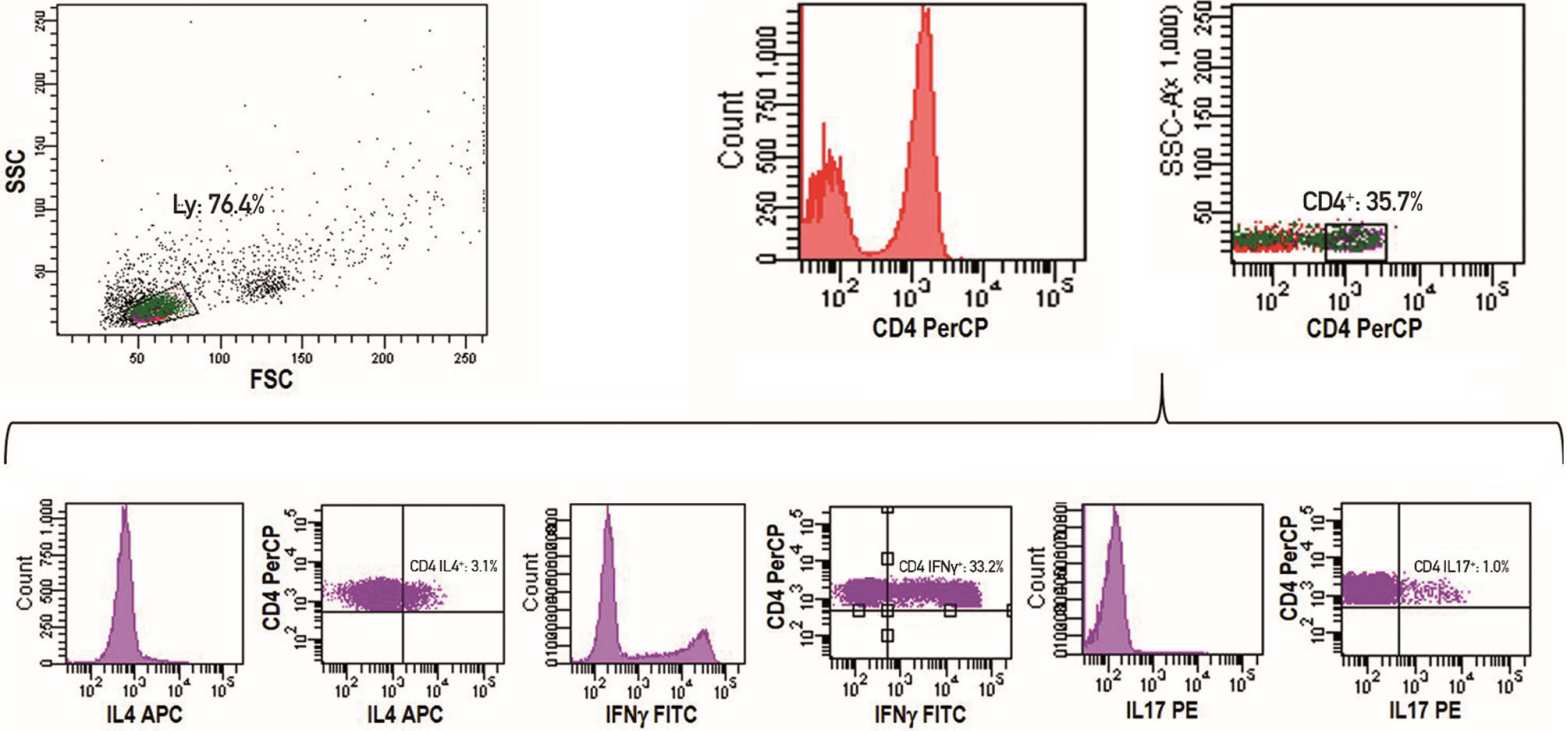
Representative example of a flow cytometry experiment. Lymphocytes are initially gated according to forward and side scatter features. An additional gate for CD4+ cells is established. The Th2, Th1 and Th17 cells results are reported as percentages of CD4+ lymphocyte population.

PB mononuclear cells (PBMC) were obtained by centrifugation on a Ficoll-Histopaque (Axis-Shield, Oslo, Norway) gradient. For intracytoplasmic detection of IFNγ, IL-4 and IL-17 producing T cells, freshly isolated PBMC were initially stimulated for 5 hours in a classic culture medium with phorbol myristate acetate PMA (50 ng/ml) (Sigma-Aldrich, St. Louis, MO) plus ionomycin (1µg/ml) (Sigma-Aldrich) in the presence of Golgi-Stop (supplemented in the Human Th1/Th2/Th17 Phenotyping Kit, Becton Dickinson (BD), San Jose, CA). Activated T cells were washed twice in PBS, divided into tubes and then fixed, permeabilized and stained using the Human Th1/Th2/Th17 Phenotyping Kit following the manufacturer’s instructions. Finally, cells were washed and analyzed by flow cytometry on a FACSCanto I flow cytometer (BD). For each analysis, 100,000 events were acquired, and analyzed using the FACSDiva software (BD), as detailed in **Figure 1**. In particular, lymphocytes were initially gated according to forward and side scatter features. An additional gate was established for CD4+ cells. The Th2, Th1 and Th17 cells results were reported as percentages of the CD4+ lymphocyte population. The absolute number of lymphocyte sub-populations was calculated by multiplying the immunophenotype percentages by the absolute number of lymphocytes obtained from the full blood count of the same sample.

### Statistical Analysis

Patients characteristics were summarized by means of cross-tabulations for categorical variables or by means of median and interquartile range (IR) for continuous variables. Non-parametric tests have been performed for comparisons between groups (Chi-Squared and Fisher Exact test in case of categorical variables, Wilcoxon and Kruskal-Wallis test in case of continuous variables). Paired samples were compared in all the different timepoints respect to the baseline (D14 *vs* D0, M8 *vs* D0, M12 *vs* D0, M18 *vs* D0, M24 *vs* D0) using the Wilcoxon test. Pearson’s correlation test was used to evaluate the association between continuous variables. The Th2/Th1 ratio levels over time was assessed using the Cochran-Mantel-Haenszel test. Receiver operating characteristic (ROC) curve was constructed to identify the best predictive Th2/Th1 ratio threshold (cut-off value) capable of correlating with clinical response to ibrutinib. All tests were 2-sided, accepting p<0.05 as indicating a statistically significant difference. All analyses were performed using the SAS software (release 9.4) and R statistical software (version 3.6.1).

## Results

### Longitudinal Analysis of T-Cell Cytokine Production Under Ibrutinib Treatment

To follow intra-individual variations, we performed a longitudinal analysis comparing paired samples from the same patient at baseline and at subsequent time points during ibrutinib treatment.

**Figure 2:**
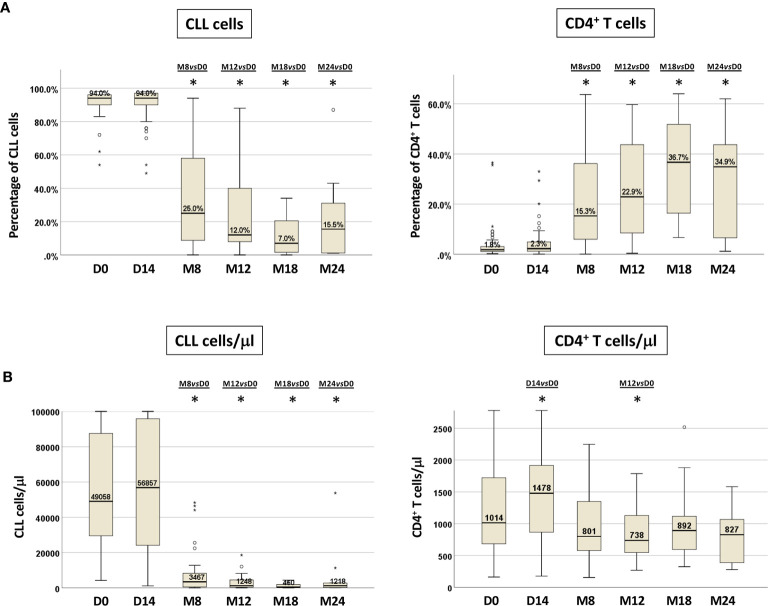
CLL cells and CD4^+^ T cells in paired PB samples before and during ibrutinib treatment. Percentage **(A)** and absolute number **(B)** of CLL cells and CD4+ T cells from paired samples included in the longitudinal analysis. Samples at D0, n=71; at D14, n=50; at M8, n=30; at M12, n=25; at M18, n=22; at M24, n=10 (Wilcoxon paired test D14 *vs* D0, M8 *vs* D0, M12 *vs* D0, M18 *vs* D0, M24 *vs* D0; *p<0.05).

We documented, as expected, a significant decrease in the percentage of CLL cells after 8 months of treatment (M8) that was maintained thereafter (Wilcoxon paired test M8 *vs* D0, M12 *vs* D0 and M18 *vs* D0, p<0.0001, each; M24 *vs* D0, p=0.005) (**Figure 2A**). Similar results were observed when analyzing the absolute number of CLL cells that showed an initial but not significant increase in the PB at D14 (**Figure 2B**).

We observed a corresponding significant rise in the percentage of CD4^+^ T cells that was retained during *in vivo* ibrutinib treatment (M8 *vs* D0, M12 *vs* D0 and M18 *vs* D0, p<0.0001, each; M24 *vs* D0 p=0.012) (**Figure 2A**, **Figure S1**). The absolute numbers of CD4^+^ T cells showed a significant increase compared to pre-treatment samples at D14 (p=0.023), followed by a subsequent decrease starting from M8, that became significant at M12 (p=0.037) (**Figure 2B**, **Figure S2**).

**Figure 3:**
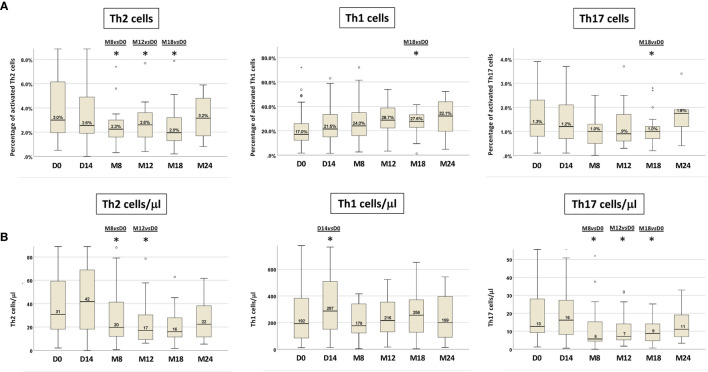
T-cell cytokine production in paired PB samples before and during ibrutinib treatment. The intra-individual variations of Th2, Th1 and Th17 activated cells are expressed as: **(A)** percentages of Th2, Th1 and Th17 cells; **(B)** absolute numbers of Th2, Th1 and Th17 cells. Samples at D0, n=71; at D14, n=50; at M8, n=30; at M12, n=25; at M18, n=22; at M24, n=10 (Wilcoxon paired test D14 *vs* D0, M8 *vs* D0, M12 *vs* D0, M18 *vs* D0, M24 *vs* D0; *p<0.05).

Furthermore, we detected a progressive increase in the percentage of CD3^+^CD4^+^IFNγ^+^ T cells (Th1), with a decrease of CD3^+^CD4^+^IL-4^+^ T cells (Th2) cells. In particular, Th1 cells increased from 17% at D0 (IR 12.1-26.2%) to 28.7% (IR 21.1-38.8%) at M12, reaching a significant difference from D0 only at M18 (p=0.03). Th2 cells were significantly reduced from M8 and remained so at M12 and M18 (M8 *vs* D0 p=0.007, M12 *vs* D0 p=0.012, M18 *vs* D0 p=0.039), while CD3^+^CD4^+^IL-17A^+^ T cells (Th17) decreased significantly only at M18 of ibrutinib treatment (p=0.006) (**Figure 3A**, **Figure S1**).

We also evaluated the variations of the Th1, Th2 and Th17 T-cell subsets in terms of absolute cell counts (**Figure 3B**, **Figure S2**). In line with the data obtained analyzing the percentages of cytokine producing T cells, a significant decrease in the absolute number of activated Th2 and Th17 cell subsets was recorded. In particular, circulating Th2 cells count diminished significantly at M8 and at M12 (p=0.019; p=0.002 respectively), while the absolute number of Th17 cells decreased significantly at M8 and was maintained up to M18 of ibrutinib therapy (M8 *vs* D0 p=0.003, M12 *vs* D0 p=0.003, M18 *vs* D0 p=0.004). On the other hand, the absolute Th1 cell count, after a significant increase at D14 of ibrutinib treatment (p=0.025), at M8 was restored as at pre-treatment conditions (D0=192/µl; M8=176/µl) with a subsequent slight increase up to M18, although not significant (**Figure 3B**, **Figure S2**).

The relative and absolute amount of CLL, CD4^+^ T cells, Th1, Th2 and Th17 cells recorded at D0 and at subsequent time points are shown in the supplementary material (**Tables S1A, B, S2A, B**).

**Figure 4:**
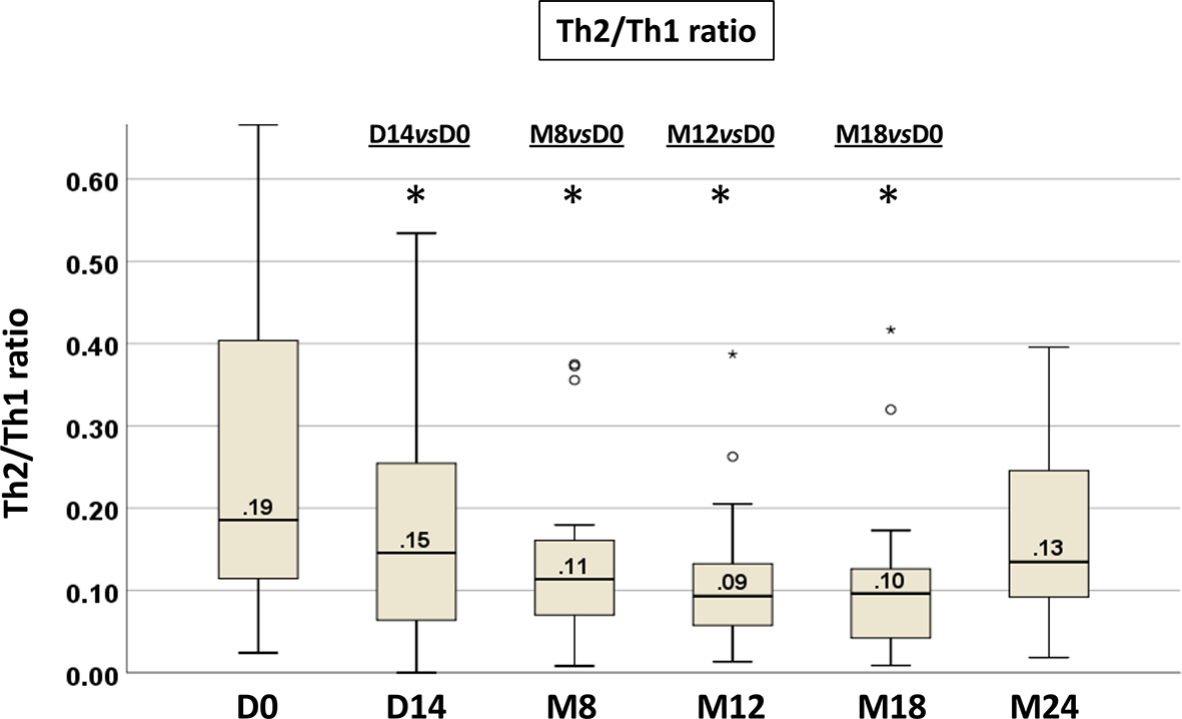
Longitudinal analysis of the Th2/Th1 ratio from paired PB samples before and during ibrutinib treatment. The intra-individual variations in T-cell production of IL-4 and IFNγ are expressed as Th2/Th1 ratio. Samples at D0, n=71; at D14, n=50; at M8, n=30; at M12, n=25; at M18, n=22; at M24, n=10 (Wilcoxon paired test D14 *vs* D0, M8 *vs* D0, M12 *vs* D0, M18 *vs* D0, M24 *vs* D0; *p<0.05).

The shift toward a type 1 or type 2 cytokine producing cells was expressed more effectively by determining the ratio of Th2 to Th1 cells, allowing us to monitor through a single value the T-cell subset modulation upon ibrutinib. The median Th2/Th1 ratio at baseline was 0.19 (range 0.02-2.59); it was <1 in 90% (n=64) and ≥1 in 10% (n=7) of cases. At the subsequent time points, the Th2/Th1 ratio showed a significant decrease already after 14 days of ibrutinib treatment, that was maintained up to M18 (Wilcoxon paired test: D14 *vs* D0 p=0.037, M8 *vs* D0 p=0.001, M12 *vs* D0 p=0.005, M18 *vs* D0 p=0.002) (**Figure 4**, **Figure S1**, **Table S1B**). At M18, 17/22 (77%) patients showed a decreased in the Th2/Th1 ratio.

To understand which of the Th1 or Th2 cells play a dominant role in the decrease of the Th2/Th1 ratio, we performed a correlation analysis of Th1 and Th2 cell modulation on the Th2/Th1 ratio at D0 and at M8 (**Tables S3A, B**). At both time points, the decrease of the Th2/Th1 ratio was sustained by the significant reduction of Th2 cells (p<0.0001 each), corresponding to a reversion of the Th2 dominant response observed in CLL.

### Modulation of T-Cell Cytokine Production, CLL Biology and Response to Ibrutinib

**Figure 5:**
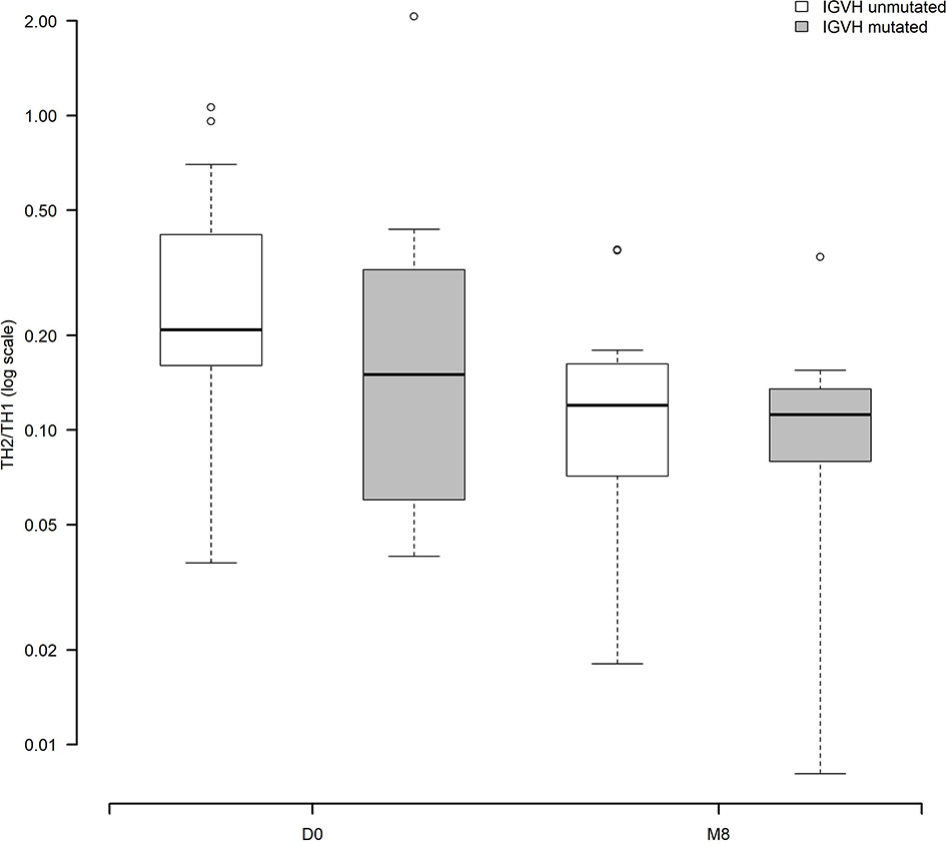
Modulation of the Th2/Th1 ratio before and during ibrutinib treatment according to the IGHV status (30 patients with paired samples, 19 with unmutated IGHV, 11 with mutated IGHV). The Cochran-Mantel-Haenszel test showed that the decrease of the Th2/Th1 ratio over time was significant only among CLL cases with unmutated IGHV genes (p<.0001).

We explored potential relationships between the modulation of Th1, Th2 and Th17 cells, and CLL clinico-biological features (**Table S4**). Interestingly, the Cochran-Mantel-Haenszel test showed that the decrease of the Th2/Th1 ratio over time was significant only among CLL cases with unmutated IGHV genes (p<.0001) in the cohort of patients with paired samples (30 patients with both D0 and M8, 19 with unmutated IGHV and 11 with mutated IGHV (**Figure 5**). These data hold true also in the total cohort of evaluated patients with paired and unpaired samples: 82 patients, 52 with unmutated IGHV and 30 with mutated IGHV (data not shown).

No significant correlations between the Th2/Th1 ratio and other clinico-biologic characteristics of CLL patients were found, possibly due to the low number of patients belonging to specific biologic subgroups (Rai stage 0-I-II [n=21] *vs*. III-IV [n= 9]; FISH del13q/+12/normal [n=23] *vs* del17p/del11q [n=7]; *TP53* wild-type [n=25] *vs* mutated [n=5]; *NOTCH1* wild-type [n=20] *vs* mutated [n=5]; *SF3B1* wild-type [n=23] *vs* mutated [n=2]; *BIRC3* wild-type [n=21] *vs* mutated [n=4]).

In order to explore a potential relationship between the modulation of Th1 and Th2 and the response of CLL to ibrutinib, we stratified the Th2/Th1 ratio at various time points according to the clinical response at M8: complete response (CR) or no complete response (no CR). This analysis showed a significant correlation between a lower Th2/Th1 ratio at baseline (p=0.05) and at M8 (p=0.023), and the achievement of a CR (**Table S5**). M18 and M24 were not included in this analysis because of the small number of samples.

**Figure 6:**
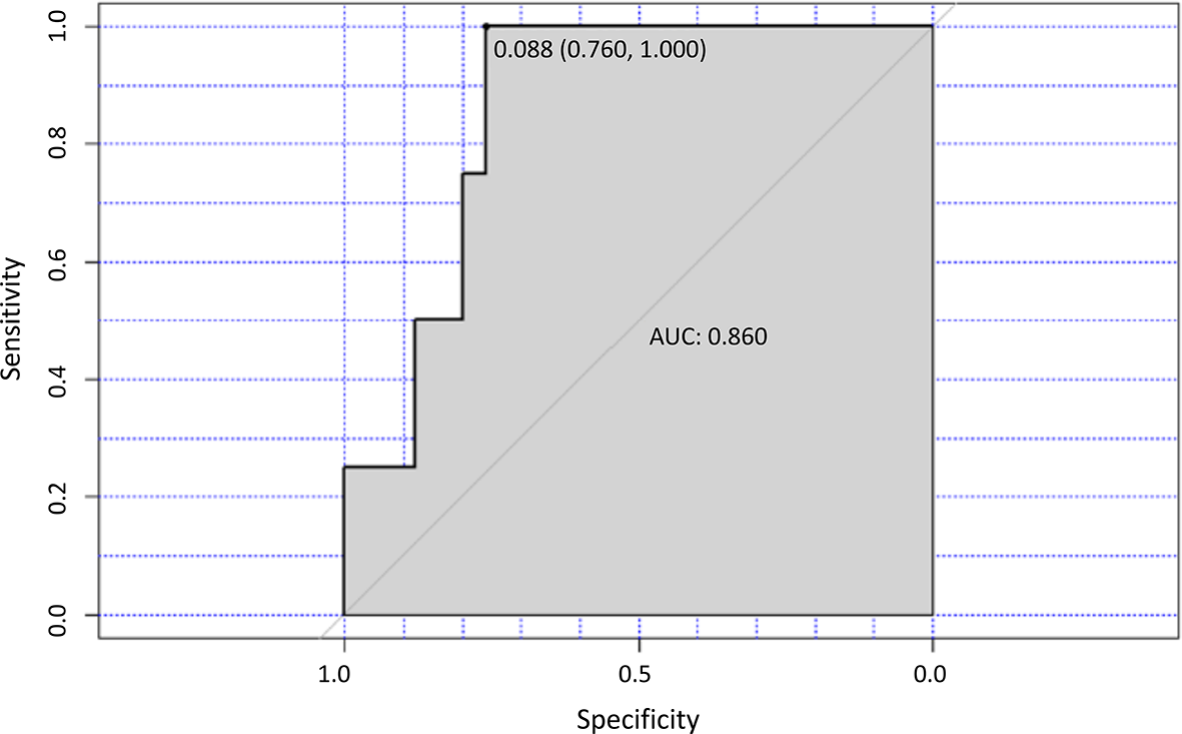
Th2/TH1 ratio and response to treatment. ROC curve analysis for the optimal cut-off point of the Th2/Th1 ratio at M8 of ibrutinib treatment. The area under the curve (AUC) value for the Th2/Th1 ratio was 0.860.

In 30 patients evaluated both at D0 and at M8, a ROC analysis showed a correlation between the decrease of the Th2/Th1 ratio below a cut-off value of 0.088 and the achievement of a CR (p=0.016) at M8 (**Figure 6**). On the basis of this cut-off, 20 of the 30 patients (66.7%) were classified as high Th2/Th1 ratio group (Th2/Th1 >0.088) and 10 (33.3%) as low Th2/Th1 ratio group (Th2/Th1 <0.088). In the low Th2/Th1 ratio group, 4 of the 10 patients achieved a CR, while no patient with a Th2/Th1 ratio over the cut-off did. The decrease of Th2/Th1 ratio <0.088 at M8 also significantly correlated with the decrease of CLL cells (p=0.025) (**Table S6**).

No correlation between Th2/Th1 modulation and the occurrence and type of infections was recorded, due to the low number of infectious events and their early occurrence in the cohort of analyzed patients.

However, a significant correlation between the Th2/Th1 decrease and an IgA increase at M12 was found (data not shown), as recently suggested (34).

## Discussion

The effect of ibrutinib on the T-cell polarization and the induced revertion of the Th2-dominant response observed in CLL has been demonstrated *in vitro* and in mouse models by Dubovsky et al. (24). A shift toward the Th1 phenotype was detected in healthy donor and CLL T cells treated with ibrutinib *in vitro*. In a mouse model of leishmania infection, treatment with ibrutinib promoted a Th1-type inflammatory response with clearance of the parasite (24).

In the present study, we evaluated by flow cytometry the modulation of CD4+ T lymphocytes in CLL patients receiving ibrutinib and rituximab front-line, thus free from the effects of previous chemotherapy. Examining the percentage and amount of Th1, Th2 and Th17 cytokine producing subsets in an analysis that followed the intra-individual variations, we documented that ibrutinib significantly shaped the T-cell profile, inducing an *in vivo* shift of the Th2/Th1 ratio, already evident after 14 days of *in vivo* ibrutinib treatment, mostly by limiting Th2 activation more than by expanding the Th1 compartment. Interestingly, the decrease of Th2/Th1 ratio over time was more prominent in the group of CLL patients with an unmutated IGHV status.

The Th1 and Th2 cell subsets in patients treated with ibrutinib have been evaluated in other studies, with some inconsistent results - mostly depending on the variability of patients’ selection and of experimental methods (i.e. plasmatic cell cytokines dosage *vs* flow cytometry) - and only partially comparable with our data (25–27). Yin et al. (26) examined 29 CLL patients under ibrutinib/ibrutinib+rituximab. They showed that the PB T-cell counts decreased and normalized under ibrutinib, in both the CD4 and CD8 subsets, at months +6 and +12 compared to the baseline. The serum concentration of a number of T-cell cytokines was measured and tended to decrease from month +3, remaining low thereafter. Interestingly, the IFNγ/IL4 concentration ratio increased after 12 months of therapy, although not significantly, and this was suggested by the authors as a surrogate marker of the reported Th1 cell expansion upon ibrutinib (24).

Niemann et al. (25) in 80 TN or relapsed/refractory (R/R) patients under ibrutinib showed a reduced expression of the exhaustion marker PD1 on T cells and a decrease of PB CD4+ and CD8+ T cells, more evident in patients with an unmutated IGHV. A global reduction of chemoattractants and inflammatory cytokines, including IFNγ and IL4, was documented in the serum from the 24^th^ week of treatment, as well as in the bone marrow (BM) supernatant.

Long et al. (27) evaluated serially collected PB samples from CLL patients treated with ibrutinib (BTK/ITK inhibitor; n = 19) or acalabrutinib (selective BTK inhibitor; n = 13) in second or further line of treatment. In contrast with the previous observations and in line with our results, ibrutinib (but not acalabrutinib) increased CD4+ and CD8+ T cells in CLL patients (more prominently in the effector/effector memory subsets), as a result of the diminished activation-induced cell death through ITK inhibition. Both agents induced a reduction in PD-1 and CTLA4 expression on T cells and of CD200 and BTLA expression, as well as of IL10 production on CLL cells, diminishing the immunosuppressive status induced by CLL cells. In contrast with our data, the authors were not able to document an *in vivo* change in T-cell polarization under ibrutinib; they showed no variation in the frequency of activated CD4+ T cells producing IFNγ, IL-4, TNFα or IL-2 from baseline to month +6, in contrast with our results.

ITK plays an important role also in regulating signaling networks downstream of the TCR that govern the differentiation of Th17 cells and Th cells producing suppressive cytokines, including Foxp3+ Treg cells and Foxp3− Type 1 regulatory T (Tr1) cells (35, 36). Recent works indicate that the absence of ITK impairs differentiation into Th17 and Tr1 cell subsets, but enhances the development of Tregs (35, 36), thus ibrutinib can likely modulate also the Th17/Tregs balance by direct inhibition of ITK (32).

However, uncertain data are present in the literature with regard to Th17 and Treg cells in CLL patients under ibrutinib (32). In our paired sample analysis, we were able to detect a significant decrease in the frequency of Th17 cells after 18 months of ibrutinib treatment and in the Th17 cells absolute count from month +8 up to month +18. In line with our results, Niemann et al. (25) documented a 22% decrease of Th17, while Long et al. documented an increase in Th17 frequency after 6 months of ibrutinib (27). A recent study documented that Tregs decreased and returned to healthy levels in CLL patients receiving ibrutinib in first-line or for relapsed/refractory disease (28); in another, the number of Tregs remained unchanged, but the Tregs/CD4+ ratio was reduced under ibrutinib treatment but not with acalabrutinib (27), leaving this field open to further investigation.

T-cell subsets could be also indirectly modulated by the reduction of the leukemic cell burden, besides the direct inhibition of ITK. Interestingly, in our study the decrease of the Th2/Th1 ratio was already induced after 14 days of *in vivo* ibrutinib treatment, well before the CLL cells start to decrease. Moreover, no linear correlation between the decrease of CLL lymphocytosis and the Th2/Th1 modulation was found by Pearson correlation analysis (data not shown). Analyzing the relationship between Th1 and Th2 modulation and disease activity, we noticed a correlation between a lower value of the Th2/Th1 ratio below 0.088 at month +8 and the achievement of a CR.

It would be of interest to expand our observations to the different CLL compartments, such as the lymph nodes, in order to clarify the global impact of ibrutinib on T cells and the CLL microenvironment, and the role of lymphocyte mobilization from lymph nodes on the documented PB changes.

Our data support the potential clinical use of ibrutinib beyond its anti-leukemic activity in CLL. Early evidences indicate that ibrutinib could be employed to control chronic graft-versus-host disease (37) and to enhance immunotherapy efficacy. Pre-clinical findings suggest that T-cell engaging CD19/CD3 bispecific antibodies against primary CLL cells from ibrutinib treated patients were more active than against samples from TN patients (38). The addition of ibrutinib to an anti-PD-L1 antibody was capable of enhancing tumor shrinkage in mice-bearing ibrutinib-resistant lymphoma, breast and colon cancer (39). Finally, ibrutinib enhanced *ex vivo* expansion, *in vivo* proliferation, and clinical activity of CAR T-cell therapy for CLL (40, 41).

In conclusion, our results translate in a clinical setting the *in vitro* and *in vivo* pre-clinical evidences provided by Dubovsky et al. on the immunomodulatory role of ibrutinib (24). We suggest that in previously untreated patients, ibrutinib may restructure the host immune surveillance by an immune subversion of the Th2 dominant response, possibly generating a host anti-tumor immune activation, that may have an impact on patients’ prognosis.

The impact of ibrutinib immunomodulation on the infectious risk and disease control needs to be confirmed on a larger series of cases.

## Data Availability Statement

The original contributions presented in the study are included in the article/**Supplementary Material**. Further inquiries can be directed to the corresponding authors.

## Ethics Statement

The studies involving human participants were reviewed and approved by the Ethic Committee of Azienda Policlinico Umberto I of Rome. The patients/participants provided their written informed consent to participate in this study.

## Author Contributions

MCP, PM, and NP performed the experiments for Th1/Th2/Th17. MCP wrote the manuscript. IDG performed the clinico-biologic correlations and contributed to write and revise the manuscript. MSDP performed flow cytometry for CLL analysis at baseline and subsequent time points. LVC contributed to data analysis and figures. LT, GR, AC, and SM enrolled and managed patients in the trial. AP and VA performed the statistical analysis. FRM wrote and run the clinical trial and enrolled patients. AG designed the study and revised the data and the manuscript. RF critically revised the manuscript. All authors contributed to the article and approved the submitted version.

## Conflict of Interest

The authors declare that the research was conducted in the absence of any commercial or financial relationships that could be construed as a potential conflict of interest.
