# Supplementary material for: TH2/TH1 Shift Under Ibrutinib Treatment in Chronic Lymphocytic Leukemia
Source: Front Oncol. 2021 Apr 15;11:637186. doi: 10.3389/fonc.2021.637186 (PMC8082026; doi:10.3389/fonc.2021.637186)

Supplementary Material

Table S1. Analysis on 208 paired PB samples before and during ibrutinib treatment. **A.** Relative amounts of Th1, Th2 and Th17 cells. **B.** Th2/Th1 ratio and relative amounts of CLL and $CD4^+$ T cells.

A.

		Percentage of Th1						Percentage of Th2						Percentage of TH17					
		D0 (n=71)	D14 (n=50)	M8 (n=30)	M12 (n=25)	M18 (n=22)	M24 (n=10)	D0 (n=71)	D14 (n=50)	M8 (n=30)	M12 (n=25)	M18 (n=22)	M24 (n=10)	D0 (n=71)	D14 (n=50)	M8 (n=30)	M12 (n=25)	M18 (n=22)	M24 (n=10)
Median		17.0%	21.5%	24.0%	28.7%	27.6%	32.1%	3.0%	2.6%	2.3%	2.6%	2.0%	3.2%	1.3%	1.2%	1.0%	0.9%	1.0%	1.8%
Interquartile range	25	12.1%	15.0%	15.8%	21.1%	21.1%	16.1%	1.9%	1.8%	1.6%	1.6%	1.2%	1.6%	0.8%	0.7%	0.5%	0.6%	0.7%	1.1%
	75	26.2%	33.4%	35.0%	38.8%	33.2%	45.6%	6.2%	5.0%	3.0%	3.7%	3.2%	5.1%	2.3%	2.1%	1.4%	1.8%	1.3%	2.3%

B.

		Th2/Th1 ratio						Percentage of CLL cells						Percentage of $CD4^+$ T-cells					
		D0 (n=71)	D14 (n=50)	M8 (n=30)	M12 (n=25)	M18 (n=22)	M24 (n=10)	D0 (n=71)	D14 (n=50)	M8 (n=30)	M12 (n=25)	M18 (n=22)	M24 (n=10)	D0 (n=71)	D14 (n=50)	M8 (n=30)	M12 (n=25)	M18 (n=22)	M24 (n=10)
Median		0.19	0.15	0.11	0.09	0.10	0.13	94.0%	94.0%	25.0%	12.0%	7.0%	15.5%	1.8%	2.3%	15.3%	22.9%	36.7%	34.9%
Interquartile range	25	0.11	0.06	0.07	0.06	0.04	0.08	90.0%	89.8%	7.6%	5.5%	1.4%	1.2%	1.1%	1.2%	6.0%	7.9%	16.1%	5.6%
	75	0.41	0.26	0.16	0.14	0.13	0.28	96.0%	97.0%	58.5%	41.0%	22.8%	34.0%	3.1%	5.0%	36.7%	45.4%	52.2%	46.6%

Table S2. Analysis on 208 paired PB samples before and during ibrutinib treatment. **A.** Absolute values of Th1, Th2 and Th17 cells. **B.** Absolute values of CLL and $CD4^+$ T cells.

A.

		Th1 cells/µl						Th2 cells/µl						Th17 cells/µl					
		D0 (n=71)	D14 (n=50)	M8 (n=30)	M12 (n=25)	M18 (n=22)	M24 (n=10)	D0 (n=71)	D14 (n=50)	M8 (n=30)	M12 (n=25)	M18 (n=22)	M24 (n=10)	D0 (n=71)	D14 (n=50)	M8 (n=30)	M12 (n=25)	M18 (n=22)	M24 (n=10)
Median		192	287	176	216	256	199	31	42	20	17	16	22	13	16	6	7	9	11
Interquartile range	25	82	146	119	118	123	87	18	18	12	9	10	10	9	8	4	5	4	7
	75	383	529	353	358	384	423	60	71	42	34	29	40	28	37	16	14	15	19

B.

		CLL cells/µl						CD4+ cells/µl					
		D0 (n=71)	D14 (n=50)	M8 (n=30)	M12 (n=25)	M18 (n=22)	M24 (n=10)	D0 (n=71)	D14 (n=50)	M8 (n=30)	M12 (n=25)	M18 (n=22)	M24 (n=10)
Median		49058	56857	3467	1248	460	1218	1024	1478	801	738	892	827
Interquartile range	25	29025	23913	493	270	116	134	684	860	573	530	583	364
	75	91471	96419	9394	4596	2160	4905	1722	1919	1393	1164	1148	1080

Table S3. Correlation analysis between the Th2/Th1 ratio and Th1 or Th2 cells at baseline (A) and after 8 months of ibrutinib treatment (B). Analysis was made by Pearson's correlation test. The values of Th2/Th1 ratio were significantly correlated at baseline and M8 to the Th2 values.

A.

	D0_Th1	D0_ Th2	D0_Th2/Th1
**D0_Th1**	1.00000	0.21233<br>0.2600	-0.34617<br>0.0609
**D0_ Th2**	0.21233<br>0.2600	1.00000	0.71452<br><.0001
**D0_Th2/Th1**	-0.34617<br>0.0609	0.71452<br>**<.0001**	1.00000

B.

	M8_Th1	M8_Th2	M8_Th2/Th1
**M8_Th1**	1.00000	0.24840<br>0.1856	-0.23784<br>0.2057
**M8_Th2**	0.24840<br>0.1856	1.00000	0.83934<br>**<.0001**
**M8_Th2/Th1**	-0.23784<br>0.2057	0.83934<br><.0001	1.00000

Table S4. Clinico-biologic features of all the evaluated patients and of those with paired samples at D0 and M8 (WT: wild-type).

**Characteristic**		**Overall N (%)**	**D0+M8 n (%)**
		71	30
Gender	Male	45 (64.3)	20 (69.0)
	Female	25 (35.7)	9 (31.0)
Binet stage	A	11 (15.5)	3 (10.0)
	B	37 (52.1)	17 (56.7)
	C	23 (32.4)	10 (33.3)
Rai stage	0	2 (2.8)	1 (3.3)
	I	14 (19.7)	7 (23.3)
	II	31 (43.7)	13 (43.3)
	III	13 (18.3)	3 (10.0)
	IV	11 (15.5)	6 (20.0)
IGHV	mutated	24 (34.3)	11 (36.7)
	unmutated	46 (65.7)	19 (63.3)
TP53	WT	57 (80.3)	25 (83.3)
	mutated	14 (19.7)	5 (16.7)
FISH	13q-	22 (31.0)	7 (23.3)
	+12	15 (21.1)	5 (16.7)
	11q-	7 (9.9)	5 (16.7)
	17p-	7 (9.9)	2 (6.7)
	No aberration	20 (28.2)	11 (36.7)
NOTCH1	WT	50 (80.6)	20 (80.0)
	Mutated	12 (19.4)	5 (20.0)
SF3B1	WT	53 (85.5)	23 (92.0)
	mutated	9 (14.5)	2 (8.0)
BIRC3	WT	57 (91.9)	21 (84.0)
	mutated	5 (8.1)	4 (16.0)

Table S5. Modulation of Th2/Th1 ratio and clinical response to ibrutinib.

		Stratified by CR		
		**NO CR**	**CR**	**p**
n		53	13	
sex (%)	M	37 (69.8)	6 (46.2)	0.201
	F	16 (30.2)	7 (53.8)	
age (median [range])		72.21 [37.19, 86.65]	72.37 [57.39, 81.24]	0.600
D0_Th2/Th1 (Median [Range])		0.20 [0.04, 2.59]	0.14 [0.02, 2.02]	**0.050**
M8_Th2/Th1 (Median [Range])		0.12 [0.02, 0.38]	0.06 [0.01, 0.09]	**0.023**

Table S6. Clinical response to ibrutinib according to the 0.088 cut-off of Th2/Th1 ratio at M8.

	M8 Th2/Th1 ratio			
	**level**	**Th2/Th1 <0.088**	**Th2/Th1 >0.088**	**p**
n		10	20	
M8_CLL cells (median [range])		0.15 [0.00, 0.30]	0.34 [0.00, 0.94]	**0.025**
CR (%)	NO CR	6 (60.0)	19 (100.0)	**0.016**
	CR	4 (40.0)	0 (0.0)	

Figure S1. CD4$^+$ T cells, Th2/Th1/Th17 cells and Th2/Th1 ratio in paired PB samples before and during ibrutinib treatment. Intra-individual variations are expressed as percentages. Samples at D0, n=71; at D14, n=50; at M8, n=30; at M12, n=25; at M18, n=22; at M24, n=10. *p<0.05.

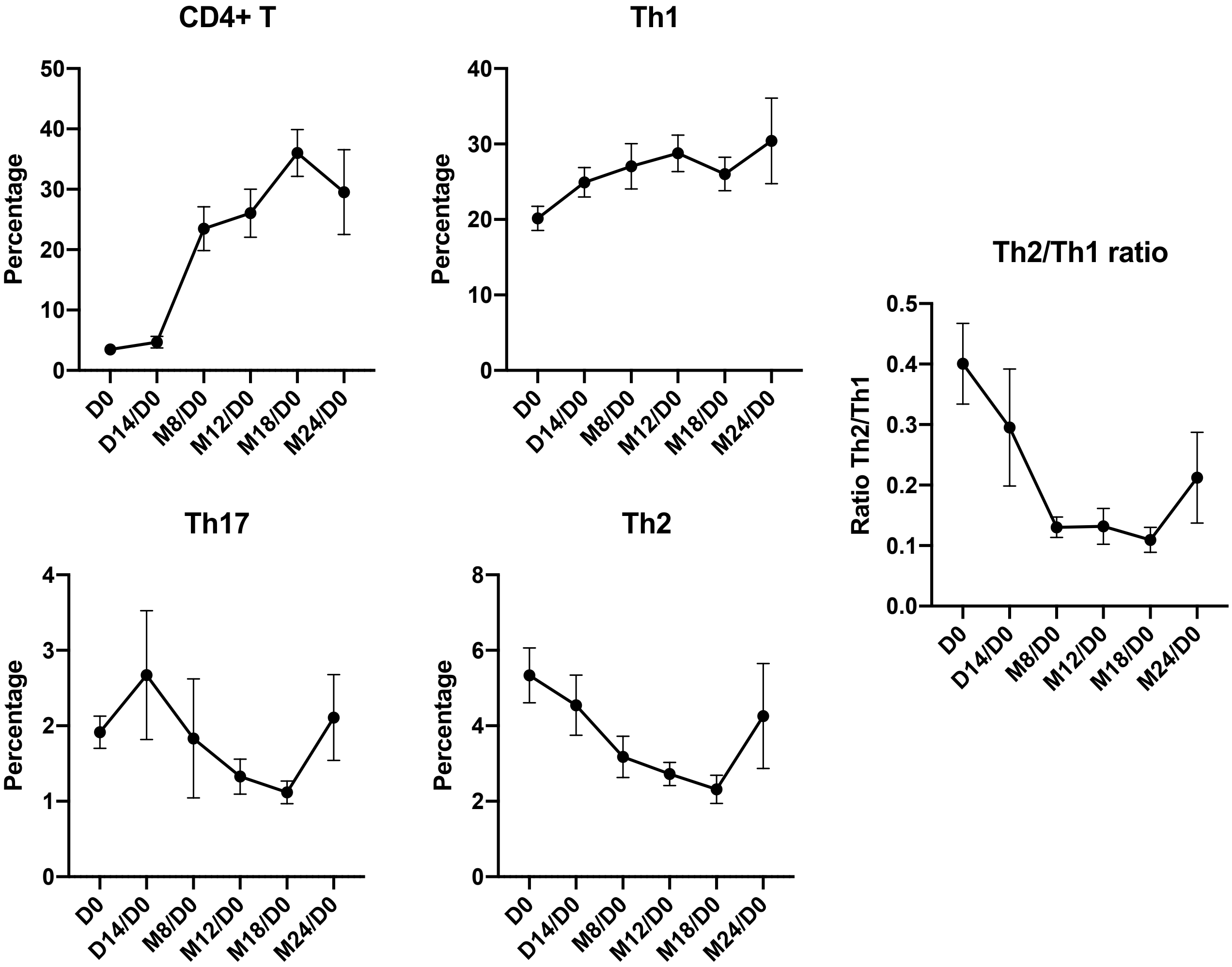

Figure S2. CD4^{+} T cells and Th1/Th1/Th17 cells in paired PB samples before and during ibrutinib treatment. Intra-individual variations are expressed as absolute values. Samples at D0, n=71; at D14, n=50; at M8, n=30; at M12, n=25; at M18, n=22; at M24, n=10.

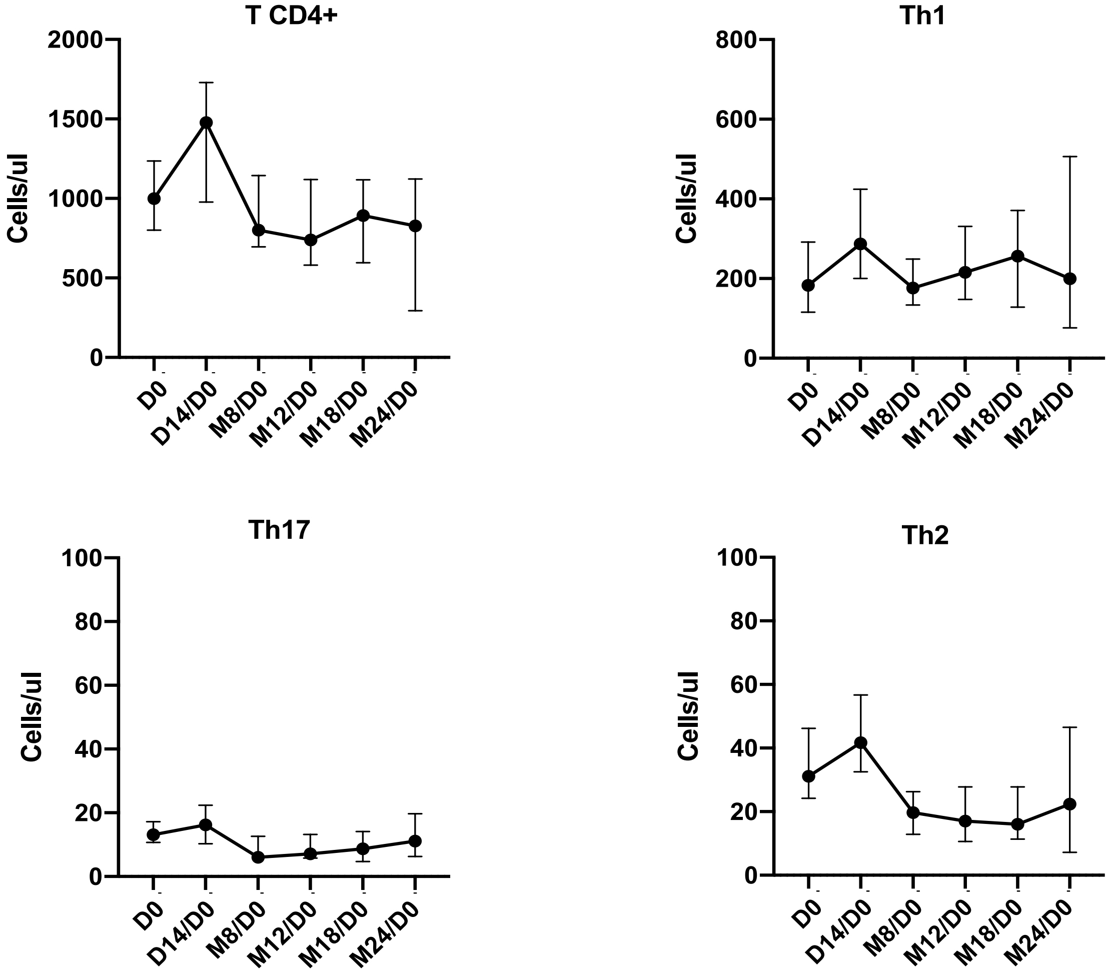

Supplement: Supplementary file 1 [file DataSheet_1.pdf]
